# Supplementary material for: Quasi-continuous melting of model polymer monolayers prompts reinterpretation of polymer melting
Source: Nat Commun. 2021 Mar 17;12:1710. doi: 10.1038/s41467-021-21799-9 (PMC7969604; doi:10.1038/s41467-021-21799-9)
Supplement: Supplementary file 1 — Supplementary Information [file 41467_2021_21799_MOESM1_ESM.pdf]

Supplementary information

Quasi-Continuous Melting of Model Polymer Monolayers Prompts Reinterpretation of Polymer Melting

Ruibin Zhang[1,2,3], William S. Fall[1,4], Kyle Wm. Hall[5], Gillian A. Gehring[4], Xiangbing Zeng[2,*], Goran Ungar[1,3,*]

1. State Key Laboratory for Mechanical Behavior of Materials, Shaanxi International Research Centre for Soft Materials, Xi'an Jiaotong University, Xi'an, 710049, China.

2. Department of Materials Science and Engineering, University of Sheffield, Sheffield S1 3JD, UK.

3. Department of Physics, Zhejiang Sci-Tech University, Hangzhou, 310018, China.

4. Department of Physics and Astronomy, University of Sheffield, Sheffield S3 7RH, UK.

5. Department of Chemistry, Temple University, Philadelphia, Pennsylvania 19122, USA.

*Correspondence to: x.zeng@sheffield.ac.uk, g.ungar@sheffield.ac.uk.

Table of Contents

1. SUPPLEMENTARY FIGURES 2

2. SUPPLEMENTARY METHODS 3

2.1. Molecular Dynamics Simulation 3

2.1.1. Simulation details 3

2.1.2. Parameter Definitions 4

2.1.2.1 Local P_2 order 4

2.1.2.2 Mobility/Positional Standard Deviation (PSD) 6

2.1.2.3 Height 6

2.1.3. Averaged 2d surface mappings 7

2.1.4. Lamellar profiles 8

2.2. Mean Field theory 10

2.3. Atomic Force Microscopy 13

3. SUPPLEMENTARY REFERENCES 13

1. SUPPLEMENTARY FIGURES

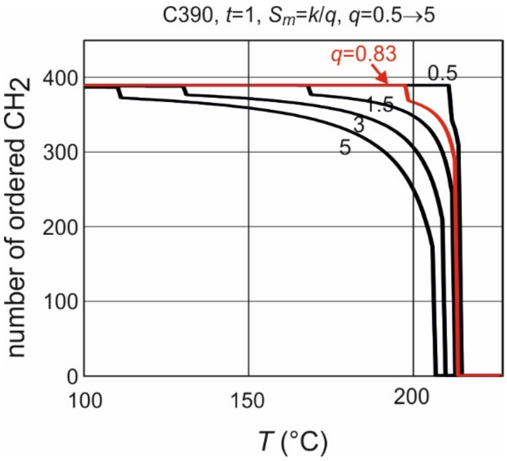

Supplementary Figure 1. The higher the melt entropy S_m, the less continuous melting. Here the same ultimate melting temperature T_m^0 of an infinitely long alkane is assumed. Note that the red curve assumes the experimental S_m value for bulk n-alkanes; nevertheless, even for such high S_m a narrow but significant range of partial melting is predicted as long as the overcrowding effect is small (t=1).

2. SUPPLEMENTARY METHODS

2.1. Molecular Dynamics Simulation

2.1.1. Simulation details

A system consisting of 1800 $C_{390}H_{782}$ chains arranged in a 6-lamella monolayer on a flat graphene surface was prepared. The SDK model represents graphene using a CG cubic lattice, so the system was constructed such that the lamellar interfaces were at a 45° with respect to the chain direction, which was parallel to underlying graphene lattice (specifically, the y direction of the simulation cell). Note that the 45° chain tilt is a result of the coarse grain nature of our MD simulations, and has been found in similar coarse-grained MD simulations previously.[S1] In particular, the polymer chains are represented using coarse-grain beads where a single coarse-grain bead represents three methylene, and graphene has been coarse-grained based on a cubic lattice approximation. For a faithful reproduction of chain tilt at lamellar surfaces, an atomic scale simulation will be needed in the future.

The system was periodic in all three directions, but the monolayer and graphene were only contiguous along x and y. Along x and y, the system was 141 nm and 282 nm, respectively. The simulations cell was 20 nm along z ensuring that the periodic images of the monolayer did not interact; the SDK model[S2] uses a 1.5 nm cut-off for non-bonded intramolecular and intermolecular interactions. In order to improve the computational efficiency of the simulations, only the positions CG beads of the alkane chains were evolved during the molecular dynamics simulations (i.e. the graphene substrate was treated as rigid).

All of the simulations conducted for this study corresponded to isothermal, isochoric (NVT) simulations, and were performed using LAMMPS.[S3] Each simulation used a timestep of 5 fs, and temperature was regulated using a four-member Nosé-Hoover[S4,S5] chain thermostat[S6] with 1-ps coupling constants. Particle positions were temporally evolved according to the velocity Verlet algorithm.[S7] Full non-bonded interactions were included for all intramole-cular neighbouring beads separated by more than two bonds (i.e., 1-4 interactions were not scaled) in accordance with the SDK model.[S2] Simulation times were

not scaled in contrast to earlier work using the SDK model to study polyethylene systems and processes.[S8,S9,S10]

The system was initially heated from 10 K to 350 K over the course of 5 ns (simulation length: 1,000,000 time steps) under constant volume conditions. The heating process was achieved by ramping the set point of the chain thermostat as internally implemented in LAMMPS[S9] via the fix NVT command.

The final configuration at 350K was then annealed at a series of different temperatures both below and at the melt temperature (484K) for a further 3,000,000 time steps and the last 51 configurations taken at 100ps intervals were used for analysis. Above the melt (>484K), the 350 K configuration was first annealed at 475 K for 2,000,000 time steps, and then annealed for a longer time at 500K for 6,000,000 time steps to simulate the melt phase. The final configuration generated from the previous run, annealed at 500K, was extended for a further 2,000,000 time steps at 500K, 550K and 600K and the last 51 configurations taken at 100ps intervals were then used for analysis.

2.1.2. Parameter Definitions

2.1.2.1 Local P_2 order

The local P_2 order parameter assesses the local alignment between individual polymer chains, and has been used extensively to distinguishing crystalline and non-crystalline polymer chain segments in polyethylene (PE) systems.[S8,S9,S10,S11,S12,S13] The P_2 value of each polymer chain bead (segment) was calculated according to $P_2(i)=(3\langle\cos^2\alpha\rangle-1)/2$, where α represents the angle between the backbone of the ith polymer bead and the backbone of a neighbouring bead. The angular brackets indicate averaging over all of the neighbouring beads within 6.1 Å of the ith bead excluding the ith bead itself and its immediate intramolecular neighbours (i.e., its bonded neighbours). Note that 6.1 Å corresponds to the approximate location of the second minimum in the bead-bead radial distribution functions for simulated monolayers considered in this study (see Supplementary Fig. 2). The backbone direction of each bead was estimated using the vector connecting its intramolecular neighbouring

beads (i.e., bonded neighbours) except for the beads at the end beads of the chain for which bond vectors were used instead. P_2 values of 1.0, 0, and -0.5 indicate that a polymer segment is parallel, randomly oriented, and perpendicular to its neighbouring segments, respectively.

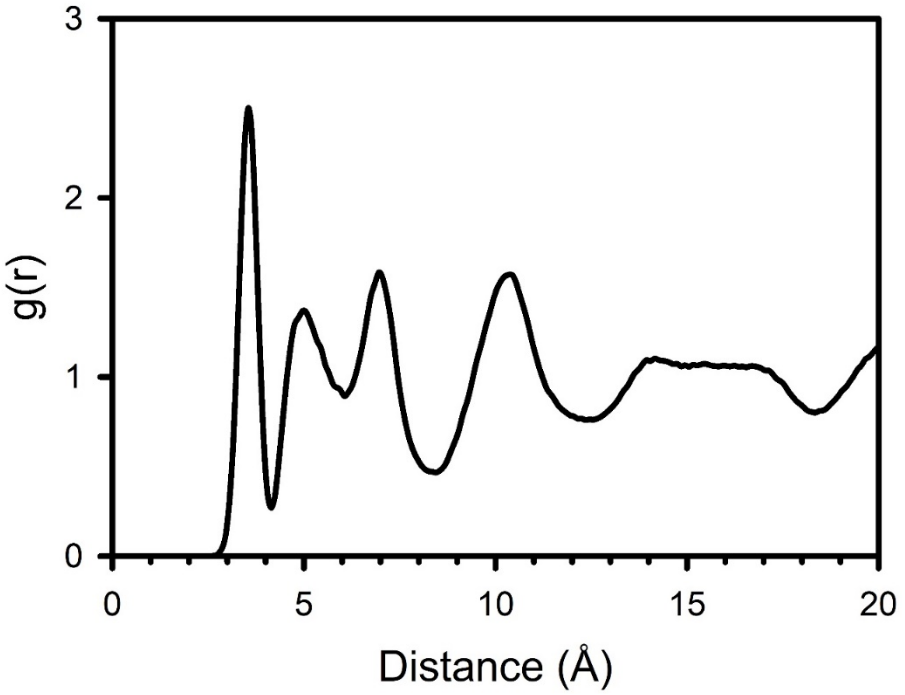

Supplementary Figure 2. The alkane bead-alkane bead radial distribution function for the final configuration of the annealing simulation at 350 K. The radial distribution was extracted using VMD,[S14] and then renormalized in accordance with the 2-D nature of the system under the assumption that the alkane beads were uniformly distributed across the underlying graphene substrate in a single layer.

2.1.2.2 Mobility/Positional Standard Deviation (PSD)

By calculating both the average squared position and average position, it is possible to quantify the movement of a given bead about its mean position throughout the simulation. The mobility (or PSD) for an arbitrary bead i, is defined as

$$PSD = \sqrt{\langle x_i^2 \rangle - \langle x_i \rangle^2 + \langle y_i^2 \rangle - \langle y_i \rangle^2 + \langle z_i^2 \rangle - \langle z_i \rangle^2}$$

Where the angle brackets indicate averaging over 51 simulation snapshots in a given 25ps time window during the simulation, corresponding to 0.5ps intervals. It may be related to the force of the AFM tip interacting with the sample by equipartition assuming that the tip is a harmonic oscillator with Hamiltonian.

$$H = \sum_i \frac{p_i^2}{2m} + \frac{k_x x_i^2}{2}$$

Where p_i is the momentum, k_x is the force constant and x_i is the position. By equipartition this is equivalent to

$$H = \langle H_{pot} \rangle + \langle H_{kin} \rangle = \frac{1}{2} k_B T + \frac{1}{2} k_B T$$

Hence we can show that the PSD^2 is proportional to the force constant at a given temperature in the simulation.

$$\frac{1}{k} \propto \frac{PSD^2}{k_B T}$$

2.1.2.3 Height

The height of a given bead is defined as the z-component of a given particles position above the surface lying in the x-y plane.

2.1.3. Averaged 2d surface mappings

Supplementary Fig. 3 shows 2d mappings of the simulation cell as viewed along the 001 direction which have been averaged from 51 instantaneous configurations taken at 100ps intervals during the simulation. Each map was calculated by partitioning the simulated surface into a 1 nm x 1 nm grid, and then locally averaging observables across 51 instantaneous configurations (at 100ps intervals). The P_2 mappings are reported in the main manuscript.

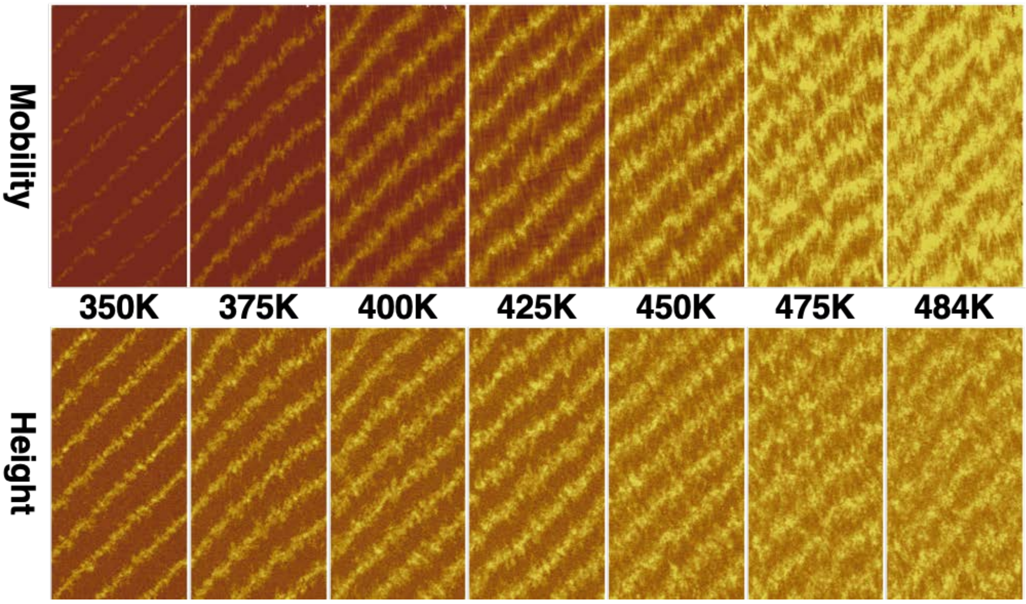

Supplementary Figure 3. Binned maps showing height and mobility for all temperatures simulated below the melt. Values calculated from MD simulation, *via* surface binning (1 nm^2) over 51 instantaneous configurations (at 100ps intervals), between 350K and 484K.

2.1.4. Lamellar profiles

Profiles (Supplementary Figs. 4-6) across the lamellae were produced by first portioning the simulation cell into 0.1nm wide strips oriented at a 45 degree to the cell walls such that the direction of the lamellae is positioned perpendicular to the strip. The averaged observables of the beads falling within the corresponding strip are then calculated by summing the observables of the beads falling inside the strip and averaging them using the strip population. This process is averaged over 51 instantaneous configurations taken at 100 ps intervals during the simulation in order to build up an averaged profile across the surface.

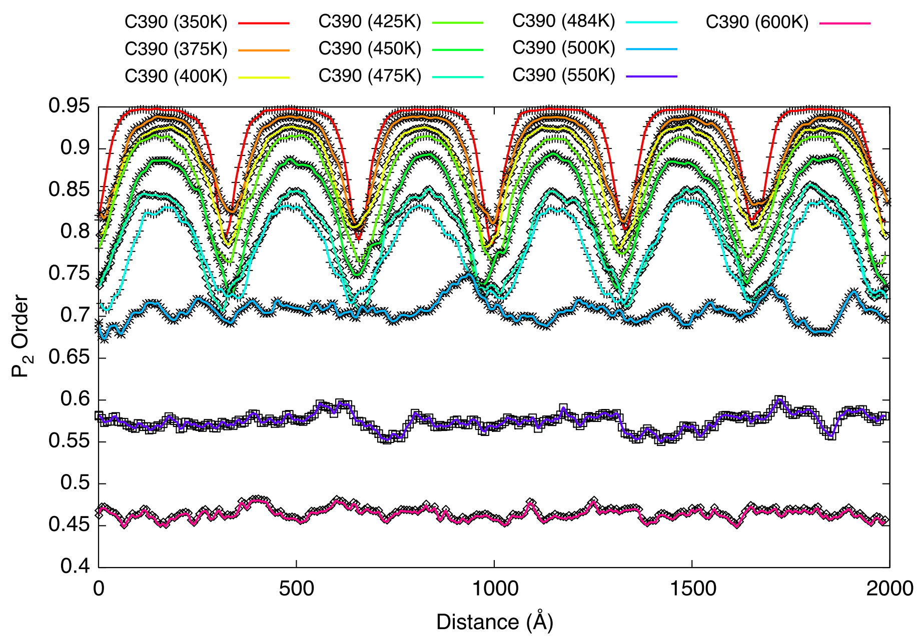

Supplementary Figure 4. P_2 profiles calculated from MD simulation, *via* surface binning (0.1 nm strips) over 51 instantaneous configurations (at 100 ps intervals), between 350K and 484K.

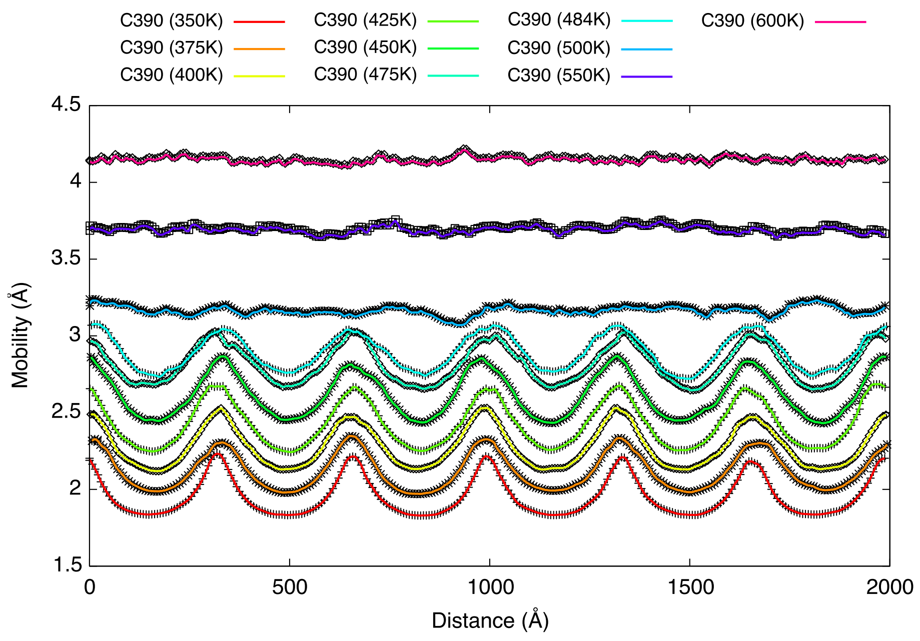

Supplementary Figure 5. Mobility profiles calculated from MD simulation, *via* surface binning (0.1 nm strips) over 51 instantaneous configurations (at 100 ps intervals), between 350K and 484K.

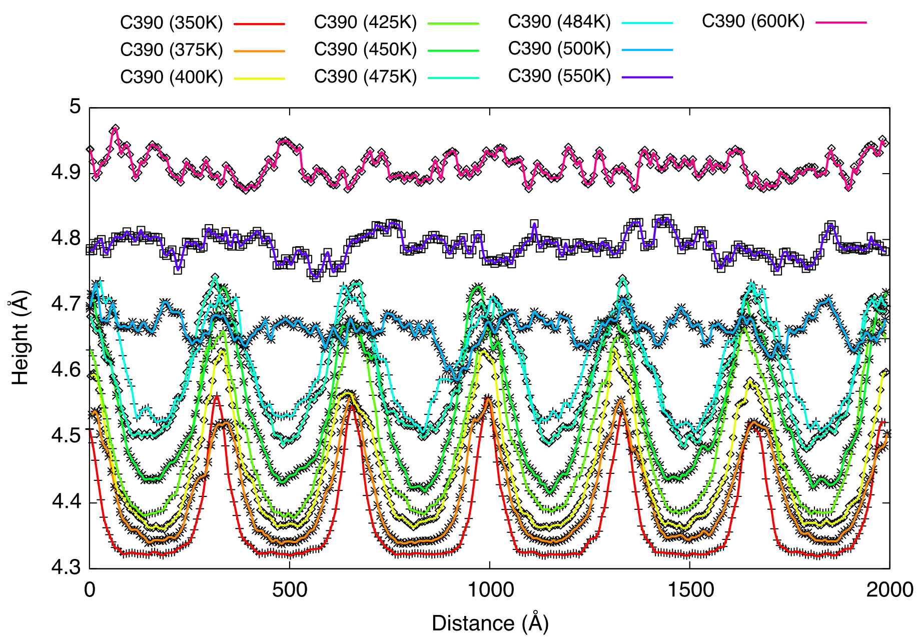

Supplementary Figure 6. Height profiles calculated from MD simulation, *via* surface binning (0.1 nm strips) over 51 instantaneous configurations (100 ps intervals), between 350K and 484K.

2.2. Mean Field theory

We take the perfectly ordered molecular chain of C_nH_{2n+1} as the reference ground state (Fig. 4a), whose enthalpy (H), entropy (S) and free energy (F) are all zero (the temperature effect, i.e. vibration of CH_2 groups are ignored). Next we consider the partially melted chain, with x disordered CH_2 groups (both ends added together), and with the remaining $n - x$ ordered CH_2 groups in the middle. In the x disordered CH_2 groups the energy and entropy levels do not jump abruptly to those of the melt immediately, instead they should gradually increase with distance from the ordered C-atoms. It is also expected that the energy increases faster initially, compared to the entropy (initial introduction of defects cost more in energy but does not increase the number of states, hence entropy, so much). In our model, we assume that the energy of disordered CH_2 groups will be that of the 2D melt, while their entropy remains as in the crystal, jumping to that of the 2D melted chain only after another t CH_2 groups. This creates a high-energy/low entropy transition layer of thickness t. t can be taken as a measure of the overcrowding effect. The existence of many other disordered chain ends close by, and the fact that all of them need to escape away from the crystalline layer (the chain ends cannot turn back into the crystalline layer), significantly reduce the number of conformations each chain end can take. It should be noted that our formulation is very similar to that previously derived by Flory *et al.*,[S15] only with a more specific evaluation of the crystal-amorphous interface free energy, i.e. the overcrowding effect.

Our model results in two possibilities as shown in Figure 5a: a smooth surface with the number of disordered surface segments, x, less than t, and a rough surface, where $x > t$.

Therefore we can write the free energy of the partially melted chain as

$$F_P(x) = \begin{cases} xH_m - k_\mathrm{B}T\ln(x+1) & 0 < x < t \\ x(H_m - TS_m) + tTS_m - k_\mathrm{B}T\ln(x+1) + 2\sigma_e & t < x < n-1 \end{cases}$$

(Equation S1)

Here H_m and S_m are the enthalpy and entropy of melting per CH_2 group, and $\sigma_e = H_e - TS_e$ is the contribution to the free energy from the two ends of each chain. The chain sliding entropy equals $k_\mathrm{B}\ln(x+1)$: for the x units of the chain

ends, there are $x+1$ choices in selecting the position at which to separate the two ends by the $n-x$ ordered units. When the surface is rough, i.e. when $x > t$, there is an additional increase in the system free energy $2\sigma_e$ due to the disappearance of the smooth end surface (the factor 2 is used as each molecule has two ends).

The minimum free energy of a partially melted chain is therefore found at

$$x = x_0 = \begin{cases} \frac{k_B T}{H_m} - 1 & 0 < x_0 < t \\ \frac{k_B T}{H_m - TS_m} - 1 & t < x_0 < n-1 \end{cases}$$

(Equation S2)

In our simple model, x_0 is independent of the length of the molecule n.

Let $H_m = T_m^0 S_m$, where T_m^0 is the ultimate melting temperature of infinitely long polymer, and Equation 2 can be rewritten as

$$x = x_0 = \begin{cases} \frac{k_B}{S_m} \cdot \frac{T}{T_m^0} - 1 & 0 < x_0 < t \\ \frac{k_B}{S_m} \cdot \frac{T}{(T_m^0 - T)} - 1 & t < x_0 < n-1 \end{cases}$$

(Equation S3)

So for a smooth surface the free energy of the most stable partially melted chain F_p is

$$F_p^{smooth} = k_B T - T_m^0 S_m - k_B T \ln\left(\frac{k}{S_m} \cdot \frac{T}{T_m^0}\right) \qquad 0 < x_0 < t$$

(Equation S4.1)

or for a rough surface

$$F_p^{rough} = k_B T - T_m^0 S_m + (t+1) T S_m - k_B T \ln\left[\frac{k_B}{S_m} \cdot \frac{T}{(T_m^0 - T)}\right] + 2\sigma_e \qquad t < x_0 < n-1$$

(Equation S4.2)

The transition from a smooth ($x_0 < t$) to a rough ($x_0 > t$) surface (T-P) happens at

$$T = T_m^0 - T_m^0 \exp[-\frac{2\sigma_e + (t+1)TS_m}{k_B T}]$$

(Equation S5)

The free energy of an n-alkane in melt (Fig. 4a) can be written as

$$F_m = n(T_m^0 – T)S_m – k_B T ln(n) + 2\sigma_e$$

(Equation S6)

Here the term $k_B T \ln(n)$ comes from the entropy due to chain sliding. Here we assume that the free energy of the ends is the same for the rough surface lamella and for the melt.

The phase transition between the partially melted chain and full melt (P-M) occurs at $F_p^{rough} = F_m$

$$\left[\left(n + t + \frac{k_B}{S_m} + 1\right) + \frac{k_B}{S_m}(\ln n - \ln \frac{T}{T_m^0 - T} - \ln \frac{k_B}{S_m})\right] T = (n+1)T_m^0$$

(Equation S7)

Alternatively the melting occurs before the surface roughens, in which case $F_p^{smooth} = F_m$

$$\left[\left(n + \frac{k_B}{S_m}\right) + \frac{k_B}{S_m}(\ln n - \ln \frac{T}{T_m^0} - \ln \frac{k_B}{S_m})\right] T = (n+1)T_m^0 + \frac{2\sigma_e}{S_m}$$

(Equation S8)

If there is no premelting at all, the direct transition from the perfectly ordered crystal (S=0) to the melt occurs at

$$\left(n + \frac{k_B}{S_m} \ln n\right) T = nT_m^0 + \frac{2\sigma_e}{S_m}$$

(Equation S9)

Comparing equations S7 - S9, it is evident that continuous partial melting will occur more easily when t is smaller, i.e. when a segment exiting the crystal is

able to attain melt-like disorder abruptly; this implies less overcrowding. The other important parameter is the melt entropy S_m. The lower the S_m, the more important the chain sliding entropy, leading to partial melting.

The parameters in our theory are estimated as follows. The heat of fusion of bulk PE is ~300 J/g, or 4.2 kJ mol^{-1}. Since T_m^{PE} = 415 K, the melt entropy in bulk is about 10 J K^{-1} mol^{-1} of CH_2. Assuming that in 2D the heat of fusion is roughly one third of that in the bulk (the number of neighbouring chains changes from 6 to 2), so H_m = 1.4 kJ mol^{-1}, and 2d melt temperature for an infinite chain T_m ~500K ($C_{390}H_{782}$ monolayer on graphite melts at 484K), S_m = 2.8 J K^{-1} mol^{-1}, or 4.7 x 10^{-24} J K^{-1} per CH_2 group, hence k_B/S_m ~ 3. The end surface free energy is chosen so that the $T_{T\text{-}P}$ is close to experimental observations, and a value of $2\sigma_e/k_B$= 430 K is used, which is 6.0 x 10^{-21} J per chain or 1.6 x 10^{-6} J cm^{-2}.

2.3. Atomic Force Microscopy

The heating stage in Cypher ES Environmental AFM is calibrated using the melting point of n-$C_{40}H_{82}$ (measured 81.0 °C, 82.0 °C in literature), benzoic acid (measured 121.0 °C, 122.3 °C in literature), and bulk $C_{390}H_{782}$ (measured 130.5 °C, 132.0 °C in literature). This is in line with calibration across the full range of temperatures by the manufacturer, with stated errors of less than 2 °C at the top surface of the stage. During the scanning, the resonance frequency of the cantilever remained constant, indicating that thermal equilibrium had been reached between the AFM probe and the sample.

3. SUPPLEMENTARY REFERENCES

S1. Milchev, A., Binder, K. Smectic C and nematic phases in strongly adsorbed layers of semiflexible polymers. *Nano Letters* **2017**, *17*, 4924-4928.

S2. Shinoda, W., DeVane, R., Klein, M. L. Multi-property fitting and parameterization of a coarse grained model for aqueous surfactants. *Mol. Simul.* **33**, 27-36 (2007).

S3. Plimpton, S. Fast parallel algorithms for short-range molecular dynamics. *J. Comp. Phys*., **117**(1), 1-19 (1995).

S4. Nosé, S. A molecular dynamics method for simulations in the canonical ensemble. *Mol. Phys.* **1984**, *52*, 255–268.

S5. Hoover, W. G. Canonical dynamics: Equilibrium phase-space distributions. *Phys. Rev. A* **1985**, *31*, 1695–1697.

S6. Martyna, G. J.; Klein, M. L.; Tuckerman, M. Nosé-Hoover chains: The canonical ensemble via continuous dynamics. *J. Chem. Phys.* **1992**, *97*, 2635–2643.

S7. Swope, W. C.; Andersen, H. C.; Berens, P. H.; Wilson, K. R. A computer simulation method for the calculation of equilibrium constants for the formation of physical clusters of molecules: Application to small water clusters. *J. Chem. Phys.* **1982**, *76*, 637-649.

S8. Hall, K. Wm., Sirk, T. W., Klein, M. L., Shinoda, W. A coarse-grain model for entangled polyethylene melts and polyethylene crystallization. *J. Chem. Phys*. **150**, 244901 (2019).

S9. Hall, K. Wm., Sirk, T. W., Percec, S.; Klein, M. L., Shinoda, W. Divining the shape of nascent polymer crystal nuclei. *J. Chem. Phys*. **151**, 144901 (2019).

S10. Hall, K. Wm., Percec, S., Klein, M. L. Polymer nucleation under high-driving force, long-chain conditions: Heat release and the separation of time scales. *J. Chem. Phys*. **150**, 114901 (2019).

S11. Yi, P., Locker, C. R., Rutledge, G. C. Molecular dynamics simulation of homogeneous crystal nucleation in polyethylene. *Macromolecules* **46**, 4723-4733 (2013).

S12. Yamamoto, T. Molecular dynamics simulation of polymer ordering. II. Crystallization from the melt. *J. Chem. Phys*. **115**, 8675-8680 (2001).

S13. Liu, C., Muthukumar, M. Langevin dynamics simulations of early-stage polymer nucleation and crystallization. *J. Chem. Phys*. **109**, 2536-2542 (1998).

S14. Humphrey, W.; Dalke, A.; Schulten, K. VMD: Visual Molecular Dynamics. *J. Molec. Graphics* **1996**, *14*, 33-38.

S15. Flory, P. J.; Vrij, A. Melting points of linear-chain homologs. The normal paraffin hydrocarbons. *J. Am. Chem. Soc.* **1963**, *85*, 3548-3553.
